# Intimate partner violence and survivor-reported partner characteristics in Ghana, Kenya, and Tanzania

**DOI:** 10.1080/16549716.2026.2664941

**Published:** 2026-04-27

**Authors:** Preeti Patel, Sentirenla Longchar

**Affiliations:** School of Computing and Digital Media, London Metropolitan University, London, UK

**Keywords:** Gender-based violence, sub-Saharan Africa, Demographic and Health Surveys (DHS), machine learning, controlling behaviours

## Abstract

**Background:**

Intimate partner violence (IPV) remains a major global public health and humanrights challenge, with a disproportionate burden in sub-Saharan Africa. While women’s experiences of IPV are well documented, fewer population-level studies examine survivor-reported partner characteristics and relational dynamics associated with perpetration.

**Objective:**

To estimate IPV prevalence in selected sub-Saharan African countries and examine demographic, socio-relational, attitudinal, and survivor-reported partnerstructural characteristics associated with IPV.

**Methods:**

We analysed data from the 2022–2023 Demographic and Health Surveys in Ghana, Kenya, and Tanzania (*n* = 59,458 ever-partnered women; 9593 reporting IPV). IPV prevalence was assessed in relation to demographic, socio-relational, and structural partnership characteristics. Random forest models were used togenerate descriptive rankings of the relative importance of selected survivor-reported partner structural characteristics across countries, with findings interpreted non-causally.

**Results:**

IPV prevalence was highest in Kenya (21.28%; 95% CI: 20.71–21.87), followed by Ghana (14.26%; 95% CI: 13.56–15.02) and Tanzania (12.34%; 95% CI: 11.66–12.99). Partner economic status and occupation consistently ranked as important structural factors. Controlling behaviours were common among women experiencing IPV, with notable cross-country variation.

**Conclusions:**

Incorporating carefully designed partner-related indicators into population-based surveys can strengthen IPV surveillance, inform targeted prevention strategies, and support gender-transformative policy in sub-Saharan Africa.

## Paper Context

- **Main findings:** Survey estimates indicate measurable intimate partner violence prevalence in Ghana, Kenya, and Tanzania, with marked cross-country differences. Emotional violence and controlling behaviours are frequently reported. Survivor-reported partner socioeconomic factors, particularly occupation and household wealth, consistently ranked among the most influential variables in the models across countries.
- **Added knowledge:** Using survivor reports of partner characteristics and behaviours, this study extends population-level IPV research beyond women’s experiences and demonstrates that structural correlates and descriptive proxies of perpetration can be examined using nationally representative survey data. Machine learning complements descriptive analyses by ranking the relative importance of selected correlated partner indicators across countries.
- **Global health impact for policy and action:** Findings highlight the value of systematically incorporating and analysing partner-related and controlling-behaviour indicators within large-scale population surveys to strengthen intimate partner violence surveillance. Improved attention to non-physical violence and structural perpetration patterns can support targeted prevention strategies, gender-transformative interventions, and context-sensitive policy development in sub-Saharan Africa.

## Background

Intimate partner violence (IPV), defined as physical, sexual, emotional, or psychological harm, inflicted by a current or former partner, remains a major global public health and human rights concern. Although IPV can be perpetrated by individuals of all genders, men constitute the majority of identified perpetrators globally, and women are disproportionately affected. Approximately 30% of women worldwide, around 736 million individuals, have experienced IPV or non-partner sexual violence in their lifetime, with more than one in four women aged 15 years and older reporting IPV [1,2].

Large-scale population surveys are central to IPV research, particularly in low- and middle-income countries (LMICs), including sub-Saharan Africa, where routine administrative data are limited. These surveys enable prevalence estimation and indirect examination of survivor-reported partner characteristics and associated risk factors [3–7]. However, underreporting, stigma, and limited access to formal support systems constrain disclosure, while provider-level barriers (e.g. time constraints and limited training) restrict screening and documentation of abusive behaviours [8,9]. Survey-based data therefore provide an important indirect window into patterns of perpetration that may not be captured through institutional reporting [4].

Research across sub-Saharan Africa and other LMIC settings identifies socio-demographic, economic, and contextual correlates of IPV perpetration, including employment instability and broader socio-cultural influences [10,11]. These determinants are commonly interpreted through ecological frameworks that situate perpetration within interacting influences at individual, relationship, community, and societal levels [12,13]. Integrating this perspective with gender theory highlights how structural inequalities and inequitable gender norms shape men’s use of violence. The concept of hegemonic masculinity underscores how dominant ideals of male authority, control, and entitlement can legitimize violence, particularly in contexts of economic strain or shifting gender roles. Household socioeconomic conditions and gendered economic dynamics are associated with both IPV perpetration and the justification of violence [14–16]. Education level and rural–urban residence further contribute to variation in perpetration patterns and normative acceptance across sub-Saharan African populations [17].

For conceptual clarity, we distinguish three related but distinct domains often grouped as ‘perpetrator characteristics’. In this study, the term ‘perpetrator characteristics’ refers to survivor-reported attributes and behaviours of husbands or partners associated with IPV, rather than directly measured perpetrator data. Structural partner attributes refer to relatively stable socio-demographic and economic positions (e.g. age, education, employment, household wealth). Relational power dynamics capture how authority and decision-making are distributed within partnerships, including gendered norms and economic dependency. Controlling behaviours refer to enacted strategies used to regulate partners’ autonomy, mobility, and social interactions. Differentiating these domains allows more precise examination of how structural positioning, power relations, and behavioural control intersect in shaping IPV perpetration in population-based data.

Beyond discrete acts of physical violence, coercive control frameworks emphasize patterned strategies through which perpetrators regulate partners’ autonomy, including restrictions on mobility, dominance in household decision-making, surveillance, and economic deprivation. Intergenerational pathways, such as childhood exposure to IPV, are associated with subsequent perpetration or victimization, suggesting the reproduction of gendered norms and behavioural repertoires over time [18–20]. Moreover, alcohol use is consistently linked to perpetration risk and severity, interacting with relational conflict and norms supportive of violence [21]. IPV during pregnancy represents a particularly harmful manifestation of control and vulnerability, documented across multiple African contexts [22].

Emerging scholarship further recognizes heterogeneity in perpetration, distinguishing situational couple violence from more chronic, coercive, and/or controlling patterns. Relational dynamics, including decision-making dominance, attitudes endorsement of violence, and patterns of control are central to understanding perpetration across contexts [23–28]. Yet, population-level research continues to prioritise women’s experiences, with comparatively limited systematic profiling of perpetrators, particularly where direct self-report data are unavailable.

In sub-Saharan Africa, gender-transformative interventions increasingly target men’s attitudes, power relations, and constructions of masculinity to prevent violence. Identifying behavioural, attitudinal, and socio-economic characteristics associated with perpetration at the population level is therefore critical. Given constraints on collecting direct perpetrator data, survivor-reported partner characteristics provide an important, though indirect, evidentiary source. Advances in data science and machine learning enable the analysis of complex, high-dimensional survey data to identify interacting predictors and rank associated factors [29,30]. Applied cautiously and transparently, these approaches can strengthen efforts to characterize structural and relational patterns associated with IPV and inform targeted prevention strategies.

In this study, we analyse data from the Domestic Violence Module of the Demographic and Health Surveys (DHS), a widely used and standardized platform for measuring IPV in population-representative samples [4,31,32]. We focus on Ghana, Kenya, and Tanzania, selected based on the availability of recent DHS domestic violence data and their representation of diverse sub-regions within sub-Saharan Africa. Drawing on women’s self-reported experiences and survivor-reported partner behaviours, we estimate IPV prevalence and examine structural partner attributes, relational power dynamics, and controlling behaviours associated with perpetration. Using machine learning approaches to assess the relative importance of selected survivor-reported partner structural factors within each country, we aim to generate population-level evidence on how structural, behavioural, and normative dimensions of associated with IPV cluster in survey data. By strengthening the empirical examination of perpetration-related patterns within DHS datasets, this analysis seeks to inform monitoring efforts and support more targeted, context-sensitive prevention strategies, while recognizing the need for complementary qualitative and contextual research to interpret patterns and guide implementation.

## Methods

### Data source and study design

This study used secondary data from the Demographic and Health Surveys (DHS), a series of nationally representative household surveys conducted under the auspices of the United States Agency for International Development (USAID). DHS surveys are standardized across countries and collect comprehensive demographic and health information using harmonized questionnaires and sampling strategies. For this analysis, we used data from the Domestic Violence Module (DVM), the primary DHS instrument for measuring IPV at the national level in low- and middle-income countries [31].

The analysis focused on three sub-Saharan African countries: Ghana, Kenya, and Tanzania that included the DVM in their most recent Phase 8 DHS surveys conducted during 2022–2023. These countries were purposively selected to enable cross-country comparison using recent, standardized data. The study population comprised women aged 15–49 years who had ever been married or partnered and who completed the DVM.

DHS surveys use a stratified two-stage cluster sampling design. The domestic violence module (DVM) is administered to a randomly selected subsample of eligible women to ensure privacy during data collection. To account for the complex survey design, analyses incorporated DHS sampling weights, primary sampling unit (cluster) identifiers, and stratification variables [32]. The analytic sample was restricted to women aged 15–49 years who were selected for and completed the Domestic Violence Module. This study employed a cross-sectional observational design.

### Definition and measurement of IPV

Intimate partner violence was defined using internationally recognized criteria consistent with guidance from the World Health Organization (WHO), the World Bank Group (WBG), and the European Institute for Gender Equality (EIGE). IPV was conceptualized as physical, sexual, emotional, or psychological violence perpetrated by a current or former spouse or intimate partner, regardless of co-residence [2,33].

Economic violence, defined as behaviours that restrict a partner’s access to financial resources, employment, or economic autonomy, is increasingly recognized as a distinct domain of IPV [34]; however, the DHS DVM does not include a standalone standardized measure of economic violence comparable to physical, sexual, or emotional violence, and it was therefore not analysed as a separate outcome in this study.

The primary outcome measure was women’s self-reported experience of physical, sexual, or emotional violence by a husband or partner. Responses across DHS items capturing these domains were combined to create a binary IPV indicator. Women were classified as having experienced IPV if they reported experiencing violence ‘yes’, ‘sometimes’, or ‘often’ for any of the relevant items. Survey weights were scaled and applied when calculating prevalence estimates and corresponding confidence intervals to account for differential probabilities of selection and non-response [32].

### Study variables

The analysis focused on a targeted subset of variables drawn primarily from the DVM, supplemented with demographic and household variables from the core DHS questionnaires. Variables were selected to capture respondent characteristics, household and partner attributes, socio-relational dynamics, and attitudes towards violence.

Demographic and economic variables included age group, education level, employment status, marital status, place of residence, household wealth quintile, and household headship. Partner-related variables included husband or partner age, education, occupation, residence status, and alcohol use. Socio-relational and attitudinal variables captured household decision-making patterns, perceptions of violence justification, economic power dynamics, childhood exposure to violence, fear of partner, and partner controlling behaviours (e.g. jealousy, monitoring movements, accusations of infidelity, and restrictions on social interactions).

A full description of study variables and their categorizations is provided in Tables 1 and 2.

**Table 1.** Study variables: demographic and economic attributes.

Variable	Categories
Ever experience of sexual violence	Yes/No
Ever experience of physical violence	Yes/No
Ever experience of emotional violence	Yes/No
**Respondent sociodemographic characteristics**	
Age group	15–19, 20–24, 25–29, 30–34, 35–39, 40–44, 45–49
Education	Primary/Secondary/Higher/None
Currently employed	Yes/No
Current marital status	Divorced/Married/Widowed/Never in union/Separated/Living with partner
Residence	Urban/Rural
**Partner and household characteristics**	
Gender of head of household	Male/Female
Wealth indicator	Poorest/Poorer/Middle/Richer/Richest
Husband/Partner age	15–95/96+/Don’t know
Husband/Partner education	No education/Primary/Secondary/Higher/Don’t know
Husband/Partner occupation	Professional/Technical/Managerial/Clerical/Sales/Agricultural – self-employed/Agricultural – employee/Household and domestic/Services/Skilled manual/Unskilled manual/Don’t know/No work
Husband/Partner currently residing with respondent	Living with him/Staying elsewhere

**Table 2.** Study variables: socio-relational and attitudinal variables.

Variable	Categories
**Household decision – making and autonomy**	
Decisions about respondents’ health, large purchases, relationships with family	Husband alone/Respondent alone/Respondent and husband/Someone else/Other
Finances	Husband alone/Respondent alone/Respondent and husband/Husband/partner has no earnings/Other
**Justification of violence**	
When wife goes out without permission, neglects the children, argue with husband, refuses to have sex, does not cook well	Yes/No/Don’t know
**Household power relations and Intimate partner risk factors**	
Respondent earns more than husband/partner	About the same/Less than him/More than him/Don’t know/Husband doesn’t bring in money
Respondent father ever beat her mother	Yes/No/Don’t know
If respondent fears her husband/partner	Most of the time afraid/Never afraid/Sometimes
Husband/partner gets jealous	Yes/No
Husband/partner monitor whereabouts	Yes/No
Husband/partner accuses respondent of unfaithfulness	Yes/No
Husband/partner restricts social interactions Husband/partner not trust with money	Yes/No Yes/No

### Data processing and management

Data processing followed DHS guidance and documentation provided by USAID and the DHS Program [32,35]. Variables requiring recoding or decoding were processed using standard DHS procedures. Columns containing entirely missing values were removed, as they did not contribute to analysis. Variables with partial missingness were retained for prevalence estimation to avoid biasing population-level estimates. Missing responses were common for some variables due to the sensitive nature of IPV-related questions. In some cases, respondents did not select predefined options such as ‘don’t know’ or ‘prefer not to say’, resulting in missing values. This non-response was treated as informative of the sensitive context rather than as random missingness, and no imputation was performed during prevalence estimation. Subgroup-specific prevalence estimates were calculated descriptively to characterize patterns of IPV across population strata and were not intended for causal inference or formal hypothesis testing.

### Random forest classification and feature importance analysis

Descriptive analyses were used to estimate weighted IPV prevalence and stratified patterns across respondent and household characteristics. To complement these results, random forest models were applied to the full analytic sample of ever-partnered women, with IPV specified as a binary outcome (‘experienced IPV’ vs. ‘did not experience IPV’). Feature importance estimates were used descriptively to compare the relative contribution of selected survivor-reported partner structural variables within the modelling framework, while accommodating potential non-linear relationships. These outputs were interpreted descriptively for feature comparison rather than causal inference or assessment of predictive performance.

Variables were intentionally restricted to a small set of survivor-reported partner structural characteristics (partner occupation, partner education, household wealth, and relative earnings within the partnership). These variables were selected because they represent relatively stable socioeconomic attributes that are consistently available across DHS datasets and can be interpreted as structural dimensions associated with IPV in this analysis. Behavioural indicators, such as partner controlling behaviours, were analysed separately using descriptive prevalence estimates among IPV survivors rather than included in the model. Partner age was initially included but excluded from the final models because it dominated impurity-based feature importance estimates, limiting the interpretability of other structural partner characteristics.

Data preprocessing was conducted to address missing values, as random forest algorithms cannot process null entries. To avoid introducing bias through imputation of substantive responses, missing values were coded as ‘Unknown’. Categorical variables were converted to numerical form using label encoding to facilitate model training. A random forest model was then trained on the processed dataset, and feature importance scores were extracted to rank partner structural characteristics according to their relative contribution within the specified model. Feature importance values reflect the relative contribution of each predictor within the model but do not indicate directionality, predictive performance, or causal effects. Rankings were therefore interpreted comparatively across variables and countries to identify prominent features rather than to estimate effect sizes. These analyses were not intended to evaluate predictive performance or to develop a classification model, but rather to provide a descriptive comparison of the relative importance of the selected variables within the specified framework.

## Results

### Sample characteristics and overall IPV prevalence

The total analytic sample comprised 59,458 ever-partnered women aged 15–49 years across Ghana, Kenya, and Tanzania. Of these, 9593 women reported experiencing physical, sexual, or emotional intimate partner violence. Country-specific weighted IPV prevalence estimates and corresponding 95% confidence intervals are presented in Table 3.

**Table 3.** Weighted prevalence of any IPV among ever-partnered women aged 15–49 years, by country.

Country	Total respondents (unweighted *n*)	Reporting any IPV (unweighted *n*)	Weighted IPV prevalence	95% CI (weighted)
Ghana	15,014	2141	14.26%	[13.56–15.02]
Kenya	29,190	5570	21.28%	[20.71–21.87]
Tanzania	15,254	1882	12.34%	[11.66–12.99]

Note: Prevalence estimates are weighted using DHS sampling weights and represent the proportion of all ever-partnered women aged 15–49 who reported experiencing IPV. Unweighted counts are presented to indicate the sample size contributing to each estimate.

Weighted estimates revealed marked cross-country variation in IPV prevalence. Kenya had substantially higher prevalence than both Ghana and Tanzania, while Ghana’s estimates were intermediate between the two. Confidence intervals showed limited overlap between Kenya and the other countries, suggesting meaningful cross-country variation in population-level IPV burden. Survey weights were applied to account for differential probabilities of selection and to enable nationally representative cross-country comparisons.

### Distribution of IPV types among survivors

Table 4 presents the distribution of emotional, physical, and sexual IPV among women who reported any IPV within each country. As shown in Table 3, 14.26% of ever-partnered women in Ghana, 21.28% in Kenya, and 12.34% in Tanzania reported experiencing any IPV; Table 4 describes the distribution of violence types within these groups of IPV survivors. Emotional violence was the most commonly reported form across all three settings, affecting a large majority of IPV survivors in Ghana and Kenya and a smaller, though still substantial, proportion in Tanzania. In contrast, physical violence was most prevalent among survivors in Tanzania, exceeding levels observed in Ghana and Kenya. Sexual violence was reported by more than one-quarter of women who experienced IPV in each country, with relatively similar prevalence across settings.

**Table 4.** Distribution of IPV types among ever-partnered women reporting any IPV, by country.

Country (*n* = women reporting any IPV)	Emotional violence % [95% CI]	Physical violence % [95% CI]	Sexual violence % [95% CI]
Ghana (*n* = 2141)	74.9 [72.66–77.41]	58.2 [55.47–60.95]	28.8 [26.32–31.25]
Kenyav (*n* = 5570)	72.5 [71.07–73.90]	72.9 [71.47–74.41]	27.4 [25.94–28.83]
Tanzania (*n* = 1882)	62.7 [59.93–65.37]	81.7 [79.54–83.92]	26.0 [23.45–28.76]

Note: Percentages are calculated among women who reported any physical, sexual, or emotional IPV (i.e. within-country IPV survivors).

Taken together, these findings indicate both shared and context-specific patterns in the composition of IPV experiences across countries and are broadly consistent with prior DHS-based regional analyses in sub-Saharan Africa, which have documented emotional violence as a frequently predominant form of IPV alongside cross-country variation in physical violence prevalence. While emotional violence predominated among survivors in all three settings, the relative prominence of physical violence varied, particularly in Tanzania, highlighting cross-country differences in the forms of IPV reported among affected women.

### Demographic, socio-relational, and attitudinal patterns

Patterns of IPV prevalence across demographic characteristics, household decision-making dynamics, attitudes towards the justification of violence, family history of violence, relative earnings, and fear of the husband or partner are summarized in Supplementary Tables S1–S4. Across countries, higher IPV prevalence was generally observed in early and mid-adulthood compared with adolescence, varied by educational attainment and rural–urban residence, and differed across marital and cohabitation statuses. Differences were also evident across household decision-making arrangements, endorsement of violence justification, relative economic position within partnerships, intergenerational exposure to violence, and reported fear of the husband or partner.

### Controlling behaviours reported by IPV survivors

To characterize relational dynamics within abusive partnerships, we examined the prevalence of selected controlling behaviours among women who reported experiencing IPV (Table 5). These estimates are descriptive and should not be interpreted as predictors of IPV occurrence. Controlling behaviours were common across all three countries, though their relative prominence varied by setting. Reports of jealousy and accusations of unfaithfulness were more frequent in Tanzania, while monitoring of women’s whereabouts was most commonly reported in Ghana. Kenya generally showed lower prevalence across several behaviours, though monitoring and jealousy were still reported by substantial proportions of IPV survivors. Restrictions on social interactions and limitations on contact with family members were comparatively less frequently reported across all settings.

**Table 5.** Descriptive prevalence of selected partner controlling behaviours among women reporting IPV, by country.

Husband/Partner behaviour	Response	Ghana % [95% CI]	Kenya % [95% CI]	Tanzania % [95% CI]
Jealous when respondent interacts with other people	No	44.12 [41.69–46.63]	29.18 [28.29–30.16]	29.44 [26.81–32.08]
	Yes	23.51 [21.87–25.17]	23.24 [22.20–24.24]	29.90 [27.29–32.60]
Monitors respondent’s whereabouts	No	40.08 [37.92–42.24]	49.27 [47.91–50.62]	40.56 [37.64–43.53]
	Yes	33.88 [31.99–36.04]	23.63 [22.70–24.58]	31.41 [28.61–34.24]
Accuses respondent of unfaithfulness	No	66.84 [63.97–69.73]	64.57 [63.04–66.19]	63.24 [60.37–66.07]
	Yes	12.80 [11.64–14.06]	8.47 [7.93–9.05]	17.72 [15.50–20.14]
Restricts respondent’s social interactions	No	74.74 [71.71–77.75]	70.87 [69.26–72.42]	76.99 [74.50–79.44]
	Yes	11.76 [10.59–13.10]	8.99 [8.33–9.65]	10.59 [8.80–12.49]
Limits respondent’s contact with family	No	87.69 [84.47–91.28]	81.68 [79.92–83.34]	82.91 [80.63–85.13]
	Yes	4.71 [3.95–5.48]	5.19 [4.74–5.68]	6.64 [5.37–8.04]

Note: Percentages are weighted estimates among women who experienced IPV in each country. Totals may not equal 100% because of missing data and/or response options beyond ‘No/Yes’.

### Structural partner characteristics and feature importance analysis

The preceding analyses describe patterns in IPV prevalence, forms of violence, and relational control dynamics reported by survivors. To further examine the relative importance of selected structural partner characteristics within the modelling framework, we applied random forest models focusing on a targeted set of survivor-reported partner socioeconomic attributes.

**Figure 1.**
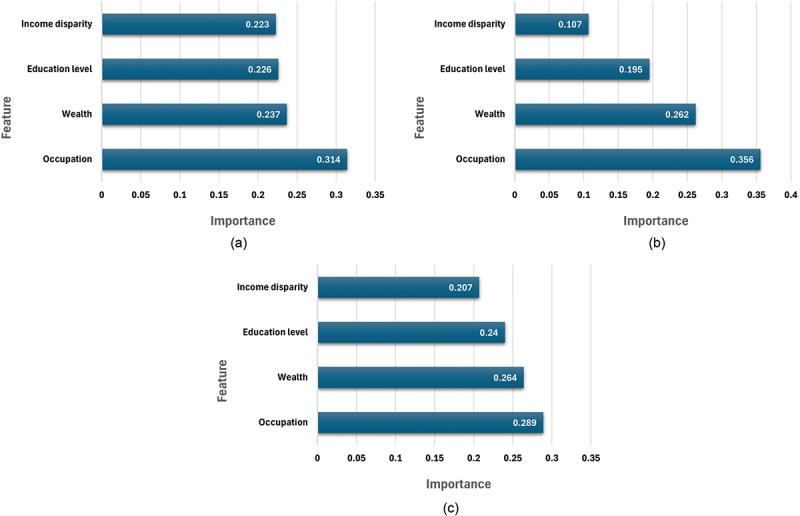
Feature importance plot of partner characteristics and relevant household indicators by country (a) Ghana, (b) Kenya, and (c) Tanzania. Note: ‘Wealth’ denotes household economic status. ‘Occupation’ and ‘Education level’ reflect the husband/partner’s job type and educational attainment. ‘Income disparity’ denotes whether the respondent earns more than her partner. Feature importance scores are relative within each country-specific model and should not be compared directly across countries.

Figure 1 presents country-specific feature importance rankings derived from random forest models examining IPV as a binary outcome using four structural partner variables: partner occupation, partner education, household wealth, and relative earnings within the partnership. Feature importance values reflect the relative contribution of each predictor to classification accuracy within the model and do not indicate direction or causal effects.

Across all three countries, the relative ordering of predictors was broadly consistent. Husband or partner occupation emerged as the most influential feature, followed by household wealth, partner education level, and income disparity between partners. Although the ranking of variables was similar across settings, the magnitude of feature importance scores varied slightly, with occupation showing the strongest relative contribution in Kenya, followed by Ghana and Tanzania. Most features exhibited importance scores within a similar range (approximately 0.20–0.30), suggesting that multiple variables contributed meaningfully within the specified model and variable set. This pattern indicates that the set of structural partner attributes included in the analysis provides informative relative rankings among the variables examined in these DHS datasets. Future research may further examine these and additional variables using similar analytical approaches.

## Discussion

This study examined IPV using a dual analytical lens: women’s self-reported experiences as survivors and survivor-reported characteristics and behaviours of husbands or partners. Drawing on recent Demographic and Health Survey (DHS) data from Ghana, Kenya, and Tanzania, the analysis provides population-level evidence on IPV prevalence and associated relational and partner-related patterns across diverse sub-Saharan African contexts. While the analysis is exploratory and limited to three countries, the findings offer insights relevant to broader regional comparisons as additional countries complete Phase 8 DHS surveys.

Across all three countries, IPV prevalence varied substantially, with higher levels observed in Kenya relative to Ghana and Tanzania. Such cross-country variation likely reflects differences in socio-economic conditions, gender norms, and contextual factors rather than a single explanatory pathway. These findings are consistent with prior DHS-based analyses and national studies documenting heterogeneity in IPV prevalence within and across sub-Saharan African settings [36–38].

### Sociodemographic and perpetration-related correlates of IPV

In line with the study’s focus on structural and relational correlates of IPV, we interpret sociodemographic characteristics not as individual risk attributes of women, but as markers of broader structural and relational conditions that shape vulnerability to IPV within partnerships.

Household economic conditions and partner employment characteristics emerged as prominent dimensions in the feature–importance analysis, indicating that these structural attributes ranked higher in relative importance within the specified model and variable set. Higher IPV prevalence was observed among women in poorer household wealth categories, consistent with evidence linking economic hardship and financial stress to increased risk of IPV perpetration [39–42]. Partner occupation also appeared relevant, with men in unstable or low-income employment more frequently associated with IPV, aligning with prior findings from sub-Saharan Africa [40].

Marital and cohabitation status further shaped IPV patterns. While IPV was commonly reported among married women, women who were separated or no longer cohabiting with partners also faced substantial risk, particularly in Kenya [36,43]. In Tanzania, elevated IPV prevalence among married and cohabiting women suggests that both formal and informal unions may expose women to violence [38]. Rural–urban differences were also observed, with higher IPV prevalence in rural Kenya and Tanzania, potentially reflecting economic dependency, limited access to support services, and entrenched gender norms. In contrast, higher urban IPV prevalence in Ghana may reflect differences in reporting, urban stressors, or shifting social norms.

### Power, control, and gender norms in intimate partner dynamics

Controlling behaviours were frequently reported among women experiencing IPV and were strongly associated with IPV across countries. Such behaviours, including jealousy, accusations of infidelity, and restrictions on movement, are widely recognized as core components of coercive control and often co-occur with physical and sexual violence [44]. Prior studies have shown that controlling dynamics are associated with increased risk of physical violence and adverse mental health outcomes among survivors [44,45].

Survivor accounts also reflect non-physical forms of coercion, including intimidation, manipulation, and cycles of affection and abuse that may foster dependency, sometimes described as traumatic bonding [46]. Perpetrators’ use of denial, minimization, justification, and blame has been documented as a common strategy to rationalize abusive behaviour and shift responsibility away from themselves [47]. These dynamics may complicate disclosure and help-seeking and influence how women perceive and report IPV in survey settings.

The specific manifestations of control varied across countries. While accusations of infidelity and jealousy were prominent predictors of IPV, reported control over women’s social interactions and family contact was comparatively lower in some contexts. In settings where extended family ties are culturally valued, controlling behaviour may be exerted more strongly through household decision-making and economic domains rather than through overt social isolation. Recent studies across sub-Saharan Africa similarly identify controlling behaviours as key predictors of IPV, while highlighting contextual variation in their expression [48].

Patterns of household decision-making further illustrate gendered power dynamics. Although joint decision-making was commonly reported across domains, men retained greater control over economic decisions, particularly in Ghana. Such gendered divisions of authority may contribute to women’s vulnerability to IPV, even in households where decision-making appears collaborative in other areas. These findings align with prior research linking male dominance in financial decision-making to increased IPV risk [26].

Notably, most women who reported IPV rejected norms justifying violence under common scenarios. This dissonance between personal beliefs and lived experiences underscores the persistence of structural constraints, economic dependency, and limited autonomy that may compel women to remain in abusive relationships despite rejecting patriarchal norms. These findings highlight the importance of distinguishing between attitudes towards violence and the structural conditions shaping women’s agency.

### Emotional and psychological abuse

Beyond physical violence, emotional and psychological abuse was highly prevalent across all three countries, with particularly high reported rates in Ghana and Kenya. Survivors often described emotional abuse, including verbal degradation, threats, and manipulation, as deeply harmful and frequently preceding physical violence. While physical and sexual IPV have received greater research attention, psychological violence remains less consistently defined and measured in both research and policy contexts [49,50].

Nevertheless, a growing body of evidence highlights the serious mental and physical health consequences of emotional abuse [51–53]. Recent studies have documented patterns of psychological violence across LMICs [54] and its associations with mental distress, coping strategies, and protective factors among specific populations [55]. Emotional violence has also been linked to adverse physical health outcomes, including hypertension [56]. A large multi-country analysis covering 53 LMICs found that while overall IPV prevalence declined between 2000 and 2021, psychological IPV increased, underscoring the need for improved measurement and recognition of non-physical forms of abuse [57].

### Economic crises, pandemics, and contextual stressors

Macro-level stressors, including economic crises and pandemics, can exacerbate IPV risk by intensifying existing socio-economic vulnerabilities. Prior research suggests that economic instability, unemployment, and disruptions to traditional provider roles may increase IPV perpetration as men seek to reassert control [58–61]. During the COVID-19 pandemic, multiple studies documented increases in IPV across diverse settings [62–69].

In contrast, the present analysis did not identify a marked increase in IPV prevalence in 2022 across the three study countries. One plausible explanation relates to the timing of data collection during the post-acute phase of the pandemic, when lockdowns had eased and immediate household stressors may have diminished. IPV occurring earlier in the pandemic may also have been underreported or less salient at the time of survey. Additionally, social protection measures and economic recovery interventions implemented in some countries may have mitigated financial stress and, by extension, IPV risk. Increased public attention to IPV during the pandemic may also have influenced reporting behaviours and perpetrator actions.

### Implications and contextual considerations

Responses to IPV often prioritize physical harm and extreme outcomes, such as homicide, partly because these are more visible and easier to document. This focus can obscure coercive control and emotional abuse, which are more difficult to define and address within legal and policy frameworks [69]. Evidence from intervention studies suggests that some perpetrator programmes improve communication and conflict-management skills, but deeper behavioural change is often constrained by distorted beliefs, emotional dysregulation, and entrenched gender norms [70,71].

Interventions shown to be effective in high-income settings, such as structured behaviour-change programmes and intensive case management, face significant challenges when transferred to sub-Saharan African contexts due to cultural norms, resource constraints, and weak legal enforcement [72–75]. Context-appropriate approaches that integrate community engagement, gender-transformative strategies, and existing health and social services may be more feasible and effective [76,77]. From a global health perspective, population-level evidence on partner-related structural and relational patterns, such as that provided in this study, can support surveillance, risk stratification, and prioritization of prevention efforts, while reinforcing the need for complementary qualitative and implementation research.

## Conclusions

This study provides recent population-level evidence on the prevalence and patterns of IPV in Ghana, Kenya, and Tanzania using nationally representative DHS data. IPV remains common across all three countries, with substantial variation in prevalence, forms of violence, and associated relational dynamics. Emotional and psychological abuse emerged as particularly prevalent, reinforcing the need for IPV prevention and response frameworks to address non-physical forms of violence alongside physical harm. By analysing survivor-reported information on partner characteristics and behaviours, this study demonstrates the value of examining structural correlates and descriptive proxies of perpetration through women’s accounts. Rather than constructing formal perpetrator profiles, this approach highlights behavioural, economic, and relational characteristics that are important in population-level analyses of IPV. The findings underscore the relevance of interventions that address controlling behaviours, economic stressors, and gendered power dynamics within intimate relationships.

From a global health perspective, integrating carefully designed partner-related indicators into large-scale surveys, such as the DHS may enhance IPV surveillance, risk stratification, and the prioritization of prevention efforts. These quantitative insights should be complemented by qualitative, contextual, and implementation research to inform culturally appropriate, gender-transformative strategies that engage both women and men. Strengthening the measurement and recognition of emotional abuse and coercive control remains critical for advancing comprehensive IPV prevention and response efforts in sub-Saharan Africa.

## Strengths and limitations

This study draws on recent, nationally representative DHS data, enabling population-level estimation of IPV prevalence and examination of relational and partner-related patterns across three sub-Saharan African countries. The use of standardized survey instruments facilitates cross-country comparability and strengthens the relevance of the findings for regional monitoring efforts.

Several limitations should be acknowledged. DHS data rely on women’s self-reported experiences, which may be affected by underreporting due to fear of retaliation, stigma, or the normalization of certain forms of violence. Survivor-reported information on partner behaviours may therefore underestimate the prevalence of some controlling or abusive practices. In addition, data collection during 2022–2023 may reflect the lingering effects of post-pandemic stressors, particularly for psychological and emotional forms of abuse, which may influence reporting. The DHS dataset also contains substantial missingness for some relational and partner interaction variables, such as household decision-making indicators, limiting the depth of analysis of relationship dynamics. Moreover, the DHS does not collect detailed information directly from perpetrators. While law enforcement or administrative records may contain richer perpetrator data, these sources primarily capture severe cases and exclude a large proportion of IPV that never reaches formal systems. As a result, this analysis necessarily relies on survivor-reported partner characteristics as indirect proxies.

Finally, the random forest feature importance analysis was used descriptively to compare the relative importance of selected variables and was not intended to assess predictive performance or classification accuracy.

Future research could benefit from applying multilevel or hierarchical modelling approaches to account for community- and regional-level clustering and to better capture structural determinants of IPV. Additional work exploring alternative machine learning techniques may also help assess the robustness of identifying IPV-related structural and relational patterns. Expanding the scope of safely collected partner-related indicators within population surveys could further strengthen IPV surveillance and research, provided ethical safeguards are maintained.

## Supplementary Material

S1 S2 S3 S4 supplementary tables.docx

## Data Availability

The data used in this study are publicly available from the Demographic and Health Surveys (DHS) Program upon registration and approval at https://dhsprogram.com/data/ available-datasets.cfm.
