# Supplementary material for: Intimate partner violence and survivor-reported partner characteristics in Ghana, Kenya, and Tanzania
Source: Glob Health Action. 2026 Apr 27;19(1):2664941. doi: 10.1080/16549716.2026.2664941 (PMC13123050; doi:10.1080/16549716.2026.2664941)
Supplement: S1 S2 S3 S4 supplementary tables.docx [file ZGHA_A_2664941_SM8680.docx]

**Table S1.** Prevalence of intimate partner violence within demographic subgroups, by country

| **Characteristic** | **Category** | **Ghana %**  **[95% CI]** | **Kenya %**  **[95% CI]** | **Tanzania %**  **[95% CI]** |
| --- | --- | --- | --- | --- |
| Age group | 15–19 | 3.89 [3.24–4.56] | 4.81 [4.41–5.23] | 4.91 [3.52–6.49] |
|  | 20–24 | 15.06 [13.77–16.35] | 15.41 [14.57–16.27] | 16.52 [14.40–19.01] |
|  | 25–29 | 17.38 [15.90–18.85] | 21.78 [20.83–22.70] | 19.38 [17.11–21.70] |
|  | 30–34 | 20.06 [18.48–21.65] | 18.53 [17.73–19.22] | 18.11 [15.81–20.25] |
|  | 35–39 | 16.94 [15.45–18.55] | 17.05 [16.29–17.88] | 14.93 [13.12–16.93] |
|  | 40–44 | 15.71 [14.40–17.37] | 12.30 [11.68–12.89] | 14.22 [12.33–16.24] |
|  | 45–49 | 10.96 [9.77–12.13] | 10.13 [9.64–10.66] | 11.93 [10.16–13.66] |
|  |  |  |  |  |
| Residence | Rural | 45.26 [43.54–47.21] | 68.24 [67.04–69.45] | 72.91 [70.04–75.49] |
|  | Urban | 54.74 [51.83–57.59] | 31.76 [30.45–33.40] | 27.09 [24.41–29.85] |
|  |  |  |  |  |
| Education | No education | 19.55 [18.19–21.00] | 6.61 [6.21–7.03] | 19.53 [17.30–21.63] |
|  | Primary | 17.27 [15.80–18.81] | 46.99 [45.88–48.24] | 65.27 [62.34–67.85] |
|  | Secondary | 56.38 [53.76–59.01] | 33.30 [32.20–34.34] | 14.61 [12.47–16.77] |
|  | Higher | 6.81 [5.91–7.78] | 13.10 [12.32–13.92] | 0.60 [0.21–1.03] |
|  |  |  |  |  |
| Employment | Yes | 85.30 [82.09–88.74] | 63.00 [61.42–64.57] | 69.55 [66.90–72.39] |
|  | No | 14.70 [13.51–16.00] | 37.00 [35.86–38.23] | 30.45 [27.82–33.20] |
|  |  |  |  |  |
| Current marital status | Married | 44.39 [42.11–46.65] | 60.19 [58.83–61.49] | 53.96 [51.20–56.81] |
|  | Living with partner | 23.68 [22.06–25.46] | 9.98 [9.32–10.65] | 23.55 [21.26–26.04] |
|  | No longer living together/separated | 9.16 [8.16–10.26] | 13.89 [13.19–14.62] | 7.08 [5.52–8.66] |
|  | Divorced | 5.34 [4.60–6.21] | 2.45 [2.13–2.80] | 11.15 [9.71–12.99] |
|  | Widowed | 2.25 [1.70–2.81] | 3.61 [3.33–3.91] | 2.81 [2.00–3.71] |
|  | Never in union | 15.18 [13.83–16.44] | 9.88 [9.30–10.51] | 1.45 [0.80–2.19] |

**Note:** Percentages represent the weighted prevalence of women reporting any physical, sexual, or emotional intimate partner violence within each subgroup. Estimates are based on nationally representative DHS data and account for complex survey design. Missing responses are excluded from subgroup–specific denominators. Results are descriptive and not intended for causal inference.

**Table S2.** Prevalence of intimate partner violence by household decision–making arrangements, by country

| **Decision domain** |  | **Decision–maker** | **Ghana %**  **[95% CI]** | **Kenya %**  **[95% CI]** | **Tanzania %**  **[95% CI]** |
| --- | --- | --- | --- | --- | --- |
| Respondent’s health |  | Husband/partner alone | 15.62 [14.32–17.04] | 11.42 [10.87–12.02] | 21.45 [19.14–23.89] |
|  |  | Respondent alone | 30.09 [28.07–31.94] | 32.71 [31.57–33.83] | 18.37 [16.08–20.90] |
|  |  | Respondent and husband/partner | 21.98 [20.56–23.60] | 25.76 [24.93–26.77] | 37.61 [34.70–40.44] |
|  |  | Someone else | 0.31 [0.11–0.53] | 0.26 [0.15–0.39] | 0.09 [0.00–0.21] |
|  |  |  |  |  |  |
| Large purchases |  | Husband/partner alone | 22.18 [20.59–23.90] | 18.57 [17.76–19.41] | 28.63 [25.97–31.20] |
|  |  | Respondent alone | 21.67 [20.05–23.16] | 15.92 [15.21–16.73] | 12.08 [10.15–14.01] |
|  |  | Respondent and husband/partner | 23.45 [22.01–25.13] | 35.49 [34.39–36.63] | 36.41 [33.77–39.37] |
|  |  | Someone else | 0.43 [0.26–0.63] | 0.06 [0.04–0.10] | 0.33 [0.10–0.63] |
|  |  |  |  |  |  |
| Relationship with family |  | Husband/partner alone | 15.88 [14.57–17.35] | 16.09 [15.25–16.90] | 27.53 [24.87–30.30] |
|  |  | Respondent alone | 22.87 [21.17–24.75] | 20.29 [19.57–21.07] | 13.66 [11.65–15.64] |
|  |  | Respondent and husband/partner | 29.08 [27.34–31.11] | 33.69 [32.57–34.85] | 36.19 [33.36–39.02] |
|  |  | Someone else | 0.15 [0.08–0.25] | 0.04 [0.01–0.07] | 0.02 [0.00–0.05] |
|  |  |  |  |  |  |
| Finance |  | Husband/partner alone | 41.40 [39.27–43.79] | 31.35 [30.25–32.47] | 34.29 [31.59–37.04] |
|  |  | Respondent alone | 7.56 [6.57–8.64] | 5.71 [5.33–6.12] | 6.44 [4.95–8.10] |
|  |  | Respondent and husband/partner | 18.07 [16.61–19.64] | 29.63 [28.66–30.64] | 35.09 [32.29–37.81] |
|  |  | Husband/partner has no earnings | 0.37 [0.21–0.59] | 1.73 [1.46–1.99] | 0.85 [0.42–1.30] |

**Note:** Percentages represent the weighted prevalence of women reporting any physical, sexual, or emotional intimate partner violence within each subgroup. Estimates are based on nationally representative DHS data and account for complex survey design. Missing responses are excluded from subgroup–specific denominators. Results are descriptive and not intended for causal inference.

**Table S3.** Prevalence of intimate partner violence by attitudes toward justification of abuse, by country

| **Scenario** | **Response** | **Ghana %**  **[95% CI]** | **Kenya %**  **[95% CI]** | **Tanzania %**  **[95% CI]** |
| --- | --- | --- | --- | --- |
| Does not seek permissions to go out | Yes | 12.28 [11.21–13.39] | 18.30 [17.52–19.12] | 39.87 [37.04–42.85] |
|  | No | 87.69 [84.31–91.19] | 81.21 [79.52–82.96] | 59.03 [56.05–61.99] |
|  | Don't know | 0.04 [0.02–0.05] | 0.49 [0.37–0.62] | 1.09 [0.49–1.90] |
|  |  |  |  |  |
| Neglects the children | Yes | 15.37 [14.12–16.53] | 28.86 [27.94–29.79] | 47.32 [44.71–50.16] |
|  | No | 84.60 [81.31–87.85] | 70.72 [69.13–72.26] | 51.99 [49.11–54.97] |
|  | Don't know | 0.03 [0.03–0.03] | 0.42 [0.30–0.58] | 0.69 [0.34–1.16] |
|  |  |  |  |  |
| Argues with husband/partner | Yes | 12.21 [11.09–13.40] | 22.84 [22.04–23.64] | 44.54 [41.52–47.29] |
|  | No | 87.74 [84.32–91.00] | 76.58 [74.87–78.28] | 54.24 [51.31–57.20] |
|  | Don't know | 0.05 [0.05–0.05] | 0.58 [0.47–0.70] | 1.22 [0.69–1.91] |
|  |  |  |  |  |
| Refuses sex | Yes | 8.72 [7.91–9.65] | 17.85 [17.15–18.56] | 33.35 [30.51–36.00] |
|  | No | 91.19 [87.85–94.34] | 80.91 [79.28–82.61] | 65.53 [62.70–68.19] |
|  | Don't know | 0.09 [0.04–0.15] | 1.24 [1.01–1.48] | 1.12 [0.55–1.82] |
|  |  |  |  |  |
| Does not cook well | Yes | 4.17 [3.62–4.77] | 8.58 [8.07–9.16] | 17.10 [14.77–19.32] |
|  | No | 95.81 [92.24–99.43] | 90.94 [89.14–92.66] | 82.09 [79.97–84.25] |
|  | Don't know | 0.03 [0.03–0.03] | 0.48 [0.36–0.59] | 0.80 [0.36–1.35] |

**Note:** Percentages represent the weighted prevalence of women reporting any physical, sexual, or emotional intimate partner violence within each subgroup. Estimates are based on nationally representative DHS data and account for complex survey design. Missing responses are excluded from subgroup–specific denominators. Results are descriptive and not intended for causal inference.

**Table S4.** Prevalence of intimate partner violence by family history of violence, relative earnings, and fear of husband or partner, by country

| **Characteristic** | **Category** | **Ghana %**  **[95% CI]** | **Kenya %**  **[95% CI]** | **Tanzania %**  **[95% CI]** |
| --- | --- | --- | --- | --- |
| Relative earnings | About the same | 3.70 [2.98–4.53] | 5.06 [4.70–5.48] | 3.74 [2.83–4.73] |
|  | Less than him | 39.97 [37.92–42.18] | 25.55 [24.53–26.49] | 23.36 [20.90–25.97] |
|  | More than him | 5.96 [5.18–6.81] | 3.99 [3.66–4.34] | 3.24 [2.33–4.31] |
|  | Husband/partner brings no income | 0.46 [0.20–0.80] | 1.70 [1.50–1.90] | 0.76 [0.32–1.28] |
|  | Don't know | 1.30 [0.90–1.79] | 1.29 [1.11–1.49] | 2.44 [1.49–3.57] |
|  |  |  |  |  |
| Father physically abused mother | Yes | 16.50 [15.15–17.94] | 42.49 [41.26–43.73] | 41.23 [38.44–43.92] |
|  | No | 79.20 [76.06–82.24] | 51.87 [50.52–53.31] | 47.67 [44.74–50.55] |
|  | Don't know | 4.30 [3.37–5.34] | 5.64 [5.25–6.08] | 11.09 [9.23–12.94] |
|  |  |  |  |  |
| Fear of husband/partner | Never afraid | 62.98 [60.13–65.66] | 51.09 [49.82–52.56] | 44.13 [41.21–47.05] |
|  | Sometimes afraid | 25.54 [23.71–27.51] | 30.48 [29.38–31.60] | 36.09 [33.30–38.91] |
|  | Most of the time afraid | 11.27 [10.33–12.24] | 18.31 [17.62–19.05] | 19.45 [17.21–21.99] |

**Note:** Percentages represent the weighted prevalence of women reporting any physical, sexual, or emotional intimate partner violence within each subgroup. Estimates are based on nationally representative DHS data and account for complex survey design. Missing responses are excluded from subgroup–specific denominators. Results are descriptive and not intended for causal inference.
